# Relationship between serum 25-hydroxyvitamin D concentrations and blood pressure among US adults without a previous diagnosis of hypertension: evidence from NHANES 2005–2018

**DOI:** 10.3389/fnut.2023.1265662

**Published:** 2023-09-28

**Authors:** Jinhang Che, Jin Tong, Xue Kuang, Caiyin Zheng, Ruoyu Zhou, Jiaqi Song, Xiaodan Zhan, Zengzhang Liu

**Affiliations:** ^1^Department of Cardiology, The Second Affiliated Hospital of Chongqing Medical University, Chongqing, China; ^2^Department of Respirology, The Second Affiliated Hospital of Chongqing Medical University, Chongqing, China

**Keywords:** 25-hydroxyvitamin D, blood pressure, hypertension, nutrition surveys, cross-sectional study

## Abstract

**Background:**

There are various cross-sectional studies that concluded that vitamin D is associated with blood pressure, but randomized controlled studies have not yielded consistent conclusions. Considering many limitations indeed, our study aimed to examine whether concentrations of 25(OH)D are inversely associated with blood pressure in people without a previous diagnosis of hypertension.

**Method:**

We analyzed data from the 2005–2018 National Health and Nutrition Examination Survey. Odds ratios (ORs) and 95% confidence intervals (CIs) were estimated by applying multivariable logistic regression models. The dose–response relationship was assessed by means of restricted cubic spline regression, and stratification analyses were employed to test the consistency between the subgroups.

**Results:**

Of 17,467 participants aged ≥ 20 years without a previous diagnosis of hypertension, 4,769 had higher blood pressure. Compared with individuals whose 25(OH)D levels were in the bottom quartile (<44.3 nnol/L), adjusting for multiple confounders, the ORs for higher blood pressure were 0.90(95%CI 0.78, 1.05), 0.85(95%CI 0.72, 0.99), and 0.86(95%CI 0.72, 1.02), respectively (*P* for trend = 0.096). Furthermore, as a continuous variable, 25(OH)D concentrations were non-linearly associated with an increased risk of hypertension (*P* < 0.001). The interaction between the sleeplessness subgroup and higher blood pressure was significant (*P* = 0.042).

**Conclusion:**

In adults without a previous diagnosis of hypertension in the United States, concentrations of 25(OH)D were inversely associated with higher blood pressure when it was <84 nmol/L.

## 1. Introduction

Approximately 1 billion individuals worldwide suffer from hypertension, making it one of the most common chronic disorders (1). Concomitantly, hypertension is an independent risk factor for cardiovascular and cerebrovascular disorders, harms quality of life, and is the leading cause of premature mortality globally (2). Nevertheless, due to the under diagnosis and under treatment of hypertension, the rates of blood pressure (BP) control remain poor and far from satisfactory worldwide. To reduce premature mortality, prevention and control of hypertension is an essential global public health strategy (3).

As a fat-soluble steroid hormone, vitamin D is essential for calcium and phosphorus balance and bone health. In addition to dietary intake, subcutaneous endogenous synthesis is an alternative major source of vitamin D. Vitamin D first must be hydroxylated to 25-hydroxyvitamin in the liver and further to 1,25-dihydroxyvitamin in the kidney enabling bioavailability (4). Interestingly, vitamin D receptors and the vitamin D enzyme system have been discovered to exist in the majority of cells in the body, with the ability to drive the transcription of hundreds of genes (5). Therefore, many scholars reasonably hypothesize that vitamin D may affect human health more than its role in bone health. To date, extensive published studies have described that vitamin D deficiency is related to osteoporosis, cancer, cardiovascular diseases, type 2 diabetes, inflammation, and autoimmune diseases (4, 5). Regretfully, large randomized controlled trials have not found convincing evidence that supplementing with vitamin D helps prevent certain disorders, such as hypertension. Moreover, existing studies have a few limitations, such as small sample sizes, insufficient adjustment of some important covariates, and specific participants. In addition, whether complications could modify the association of interest remains unclear.

In the current study, we attempted to investigate the relationships between serum 25(OH)D and BP, detailing systolic and diastolic blood pressure, in a nationally representative sample of US adults with no previous diagnosis of hypertension.

## 2. Materials and methods

### 2.1. Study population

The National Health and Nutrition Examination Survey (NHANES) is a stratified, multistage probability survey, which collected health and nutrition information among the non-institutionalized US population through interviews, physical examination, and laboratory testing. The National Center for Health Statistics of the Centers for Disease Control and Prevention (CDC) conducted NHANES, which was approved by the Institutional Review Board of the National Center for Health Statistics. All participants or their guardians provided signed informed consent.

**Figure 1:**
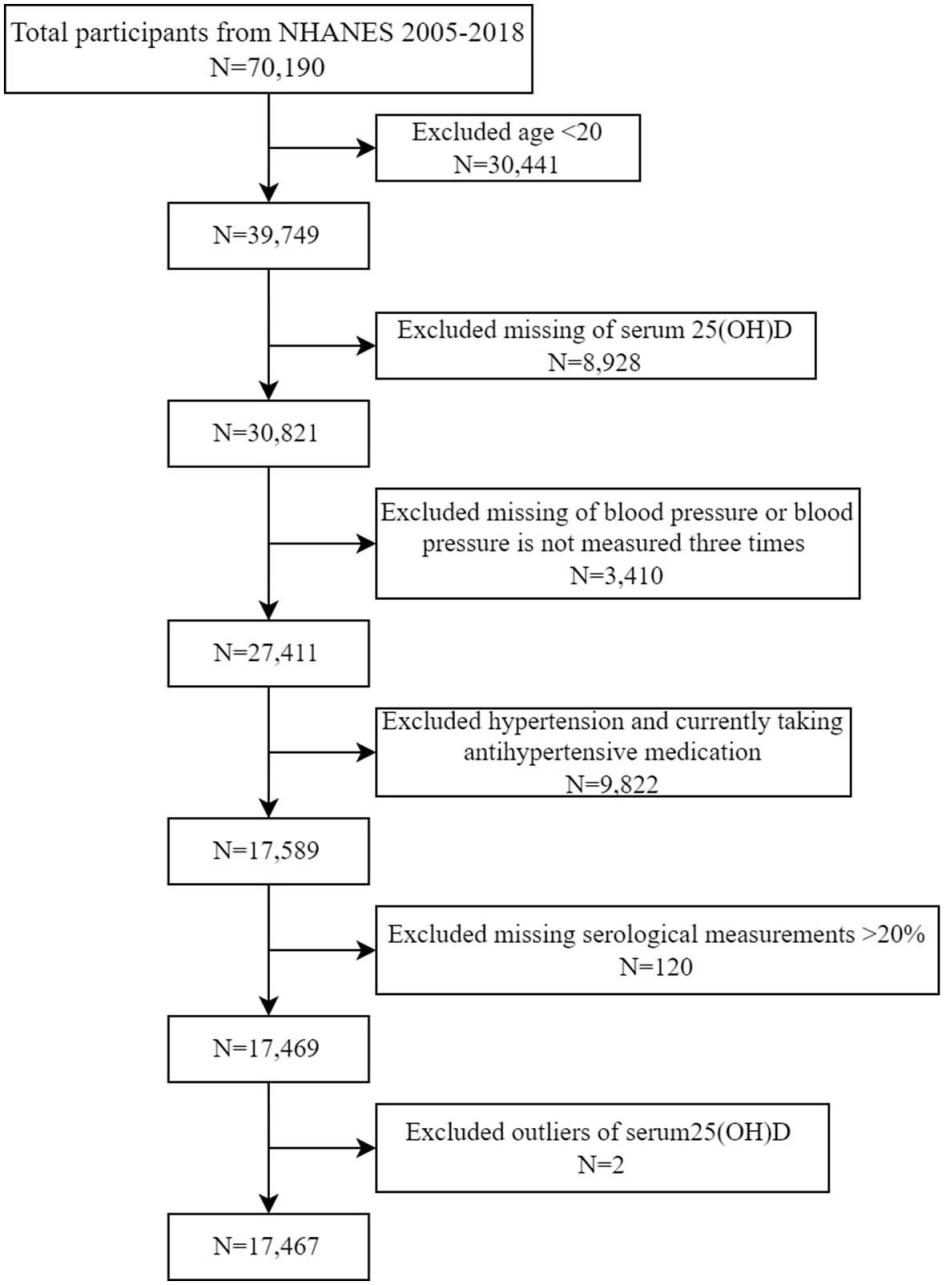
Participants inclusion flowchart.

In the current study, we used data from seven consecutive NHANES 2-year cycles (2005–2006, 2007–2008, 2009–2010, 2011–2012, 2013–2014, 2015–2016, and 2017–2018). Individuals (aged ≥ 20 years) with no previous diagnosis of hypertension were included in the analysis. We excluded participants with missing blood pressure (BP) or serum 25(OH)D measurements, with measurements of BP <3 times, and with missing serological values >20%. Outliers (through the “scatter” command in Stata) of serum 25(OH)D were further excluded. Hypertension was defined as a self-reported doctor diagnosis of hypertension and use of antihypertensive drugs. We finally had 17,467 eligible participants enrolled for analyses (Figure 1).

### 2.2. Measurement of serum 25(OH)D

In the NHANES 2005–2006, serum 25(OH)D was measured by the DiaSorin assay kit [DiaSorin Corporation 25(OH)D 125I RIA kit; DiaSorin Corporation, Stillwater, Minnesota, USA]. Beginning from the 2007–2008 cycle, serum 25(OH)D concentrations were tested by a standardized liquid chromatography-tandem mass spectrometry (LC-MS/MS). The regression approach was used to convert serum 25(OH)D data from the NHANES 2005–2006 to equivalent 25(OH)D measurements using the LC-MS/MS method. We used LC-MS/MS-equivalent data for all analyses as recommended by the CDC. Detailed information is available at https://wwwn.cdc.gov/nchs/nhanes/vitamind/analyticalnote.aspx.

### 2.3. Measurement of BP

Three successive blood pressure readings were taken after 5 min of calmly resting in a sitting position and establishing the maximum inflation level (MIL). A fourth try would be performed if the measurement of BP was interrupted or incomplete. All measurements of BP were taken in the mobile examination center (MEC). The absolute BP was obtained by averaging three valid measurements. Participants were categorized as having higher BP if systolic blood pressure (SBP) ≥130 mmHg or diastolic blood pressure (DBP) ≥80 mmHg.

### 2.4. Covariates

Several covariates were evaluated as potential confounding factors. Information on age (years), sex (male and female), race (non-Hispanic white, non-Hispanic black, Mexican American, and other race), education level (less than high school, high school, and college education or above), smoking status (current smoking, previous smoking, and never smoking), heavy alcohol drinking (yes and no) was acquired through household interviews utilizing standardized questionnaires. Current smoking was defined as smoking at least 100 cigarettes in one's life and still smoking, previous smoking as smoking at least 100 cigarettes in one's life but not presently smoking, and non-smoking as smoking fewer than 100 cigarettes in one's life. Alcohol consumption was estimated based on self-reported data on the average number of daily drinks. Heavy alcohol drinking was defined as having more than one drink per day for women and more than two drinks per day for men, on average, over the previous 12 months. Vitamin D supplementation refers to the intake of vitamin D2 + D3 in the previous month. During the mobile examination center visit, physical measurements such as body mass index (BMI) and waist circumference were collected. BMI was computed by dividing weight in kilos by height in meters squared.

In addition, serum samples from participants were obtained during the medical examination and frozen and stored at temperatures below −70°C until analysis. Serum concentrations of calcium, alanine aminotransferase (ALT), aspartate aminotransferase (AST), creatinine, albumin, triglyceride, cholesterol, total bilirubin, blood urea nitrogen (BUN), uric acid (sUA), and HbA1c were included at the baseline. The Chronic Kidney Disease-Epidemiology Collaboration (CKDEPI) (6) equation was used to compute the mean estimated glomerular filtration rate (eGFR, ml/min/1.73 m^2^). According to the KDOQI (7) guidelines, CKD can be classified into five stages: CKD1 (eGFR ≥ 90), CKD2 (60 ≤ eGFR < 90), CKD3 (30 ≤ eGFR < 60), CKD4 (15 ≤ eGFR < 30), and CKD5 (eGFR < 15).

At the baseline, comorbidities consist of a history (yes, no) of self-reported physician-diagnosed diabetes, coronary artery disease (CAD), stroke, cancer, and sleeplessness.

### 2.5. Statistical analysis

Given the complex sampling design of the NHANES, all analyses were properly weighted in accordance with the analytical guidelines from the National Health and Nutrition Examination Survey for the years 2005 through 2018, which is accessible at https://wwwn.cdc.gov/Nchs/Nhanes/AnalyticGuidelines.aspx#print. As mentioned before, we combined seven consecutive 2-year cycles (2005–2006, 2007–2008, 2009–2010, 2011–2012, 2013–2014, 2015–2016, and 2017–2018) to estimate with greater precision and smaller sampling error.

A complex weighting method was utilized for the data description and statistical analysis in this study by using the “Survey” package in R software. The categorical variables were described as frequencies (unweighted) and proportions (weighted), whereas the continuous variables were described as mean standard deviation. The differences were tested using the Wilcoxon rank-sum test for continuous variables and the chi-square test with Rao–Scott second-order correction for categorical variables. To investigate the association between serum 25(OH)D and higher BP, serum 25(OH)D levels were divided into quartiles, and adjusted ORs for increased blood pressure were calculated through weighted logistic regression in three distinct models. Model 1 was unadjusted. Model 2 was adjusted for age, gender, and ethnicity. Model 3 was adjusted for age, gender, ethnicity, education level, smoking status, heavy alcohol drinking, vitamin D supplementation, BMI, waist circumference, diabetes, coronary heart disease, stroke, cancer, sleeplessness, CKD, serum calcium, ALT, AST, Scr, albumin, triglyceride, cholesterol, total bilirubin, BUN, sUA, and HbA1c. The lowest quartile of serum 25(OH)D was used as the reference group. To further examine the dose–response relationship between serum 25(OH)D concentrations and BP, restricted cubic spline regression was performed with four knots, with the multivariate adjustment in Model 3. If the *p*-value for non-linearity was >0.05, we considered the dose–response relationship to be linear, otherwise non-linear. Stratification analyses were performed to examine whether the correlations differed for subgroups classified comorbidities including diabetes, CAD, stroke, cancer, CKD, and sleeplessness.

A two-sided *p* < 0.05 was used to determine statistical significance. All the data analyses were performed using the software Stata/SE V.15.1 (StataCorp LLC, College Station, TX, USA) and R software 4.2.2 (http://www.R-project.org).

## 3. Results

### 3.1. Baseline characteristics

Table 1 displays a broad overview of the weighted features of all 17,467 study participants according to serum 25(OH)D quartiles (Q1 < 44.3, 44.3 ≤ Q2 < 60.5, 60.5 ≤ Q3 < 77.7, and Q4 ≥ 77.7). Among the 17,467 adults (48.7% were men and 51.3% were women) without a previous diagnosis of hypertension. The average age was 42.8 years old; 65.7% were non-Hispanic white, 10.1% were Mexican American, 9.3% were non-Hispanic black, and 14.9% were “Other” races. Approximately 63.6% attained an educational level greater than a high school diploma. Across quartiles of serum 25(OH)D, significant differences (*p* < 0.05) were found in smoking status, heavy alcohol drinking, vitamin D supplementation, BMI, waist circumference, cancer, sleeplessness, CKD, serum calcium, ALT, AST, Scr, albumin, triglyceride, cholesterol, total bilirubin, BUN, sUA, and HbA1c. Participants with higher BP were 4,769, and the weighted percentage reached 25%. Of these, 3,382 patients suffered from higher systolic blood pressure, and 2,739 suffered from higher diastolic blood pressure.

**Table 1:** Characteristics of the study participants by baseline serum 25(OH)D quartiles.

**Characteristics**	**Serum 25(OH)D, nmol/L**					***p*-value**
	**Overall**	** <44.3**	**44.3– <60.5**	**60.5– <77.7**	**≥77.7**	
Unweighted, n	17,467	4,349	4,379	4,366	4,373	
Age, year	42.8 ± 15.6	38.4 ± 14.3	39.7 ± 14.3	42.5 ± 15.2	47.3 ± 16.3	<0.001
Sex, (%)						<0.001
Male	8,605 (48.7%)	2,075 (47.9%)	2,334 (54.5%)	2,300 (53.3%)	1,896 (41.6%)	
Female	8,862 (51.3%)	2,274 (52.1%)	2,045 (45.5%)	2,066 (46.7%)	2,477 (58.4%)	
Ethnicity, (%)						<0.001
Non-Hispanic white	7,085 (65.7%)	687 (31.0%)	1,357 (54.4%)	2,101 (72.0%)	2,940 (86.3%)	
Mexican American	3,014 (10.1%)	937 (18.5%)	1,019 (16.0%)	731 (8.8%)	327 (2.9%)	
Non-Hispanic black	3,012 (9.3%)	1,611 (29.2%)	691 (9.6%)	416 (4.5%)	294 (2.4%)	
Others	4,356 (14.9%)	1,114 (21.2%)	1,312 (20.0%)	1,118 (14.6%)	812 (8.3%)	
Education level, (%)						<0.001
< High school	3,958 (14.5%)	1,064 (19.5%)	1,169 (17.8%)	987 (14.3%)	738 (9.8%)	
High school graduate	3,828 (21.8%)	1,070 (25.4%)	940 (22.3%)	938 (21.5%)	880 (20.0%)	
College education or above	9,667 (63.6%)	2,210 (55.0%)	2,266 (59.8%)	2,440 (64.2%)	2,751 (70.1%)	
Smoking status, (%)						<0.001
Never smoking	10,277 (58.3%)	2,666 (60.4%)	2,652 (59.9%)	2,485 (55.8%)	2,474 (58.1%)	
Previous smoking	3,488 (21.5%)	611 (14.4%)	811 (18.7%)	978 (24.2%)	1,088 (25.0%)	
Current smoking	3,693 (20.1%)	1,067 (25.1%)	914 (21.3%)	902 (20.0%)	810 (16.8%)	
Heavy alcohol drinking, (%)	6,338 (39.1%)	1,639 (39.7%)	1,589 (39.0%)	1,610 (40.0%)	1,500 (38.3%)	<0.001
Vitamin D supplementation, (%)	5,765 (36.9%)	528 (12.2%)	1,043 (24.7%)	1,647 (35.7%)	2,547 (59.0%)	<0.001
BMI, kg/m^2^	28.0 ± 6.3	29.7 ± 7.7	28.9 ± 6.4	28.0 ± 6.1	26.5 ± 5.2	<0.001
Waist circumference, cm	96.3 ± 15.7	99.0 ± 18.0	98.3 ± 15.9	96.5 ± 15.2	93.3 ± 14.1	<0.001
Comorbidity, %						
Diabetes	1,096 (4.4%)	246 (4.5%)	276 (4.5%)	277 (4.4%)	297 (4.2%)	0.700
CAD	258 (1.3%)	49 (1.2%)	54 (1.2%)	61 (1.1%)	94 (1.6%)	0.328
Stroke	235 (1.0%)	60 (1.2%)	45 (0.8%)	49 (0.9%)	81 (1.2%)	0.473
Cancer	1,103 (7.3%)	142 (4.2%)	201 (4.5%)	270 (6.6%)	490 (11.4%)	<0.001
Sleeplessness	3,517 (22.9%)	735 (18.7%)	773 (19.6%)	878 (22.7%)	1,131 (27.5%)	<0.001
CKD						<0.001
CKD1	12,371 (68.7%)	3,585 (81.9%)	3,401 (77.7%)	2,989 (69.1%)	2,396 (55.4%)	
CKD2	4,493 (28.4%)	685 (16.5%)	883 (20.7%)	1,234 (28.7%)	1,691 (39.7%)	
CKD3	572 (2.8%)	70 (1.4%)	92 (1.6%)	138 (2.1%)	272 (4.8%)	
CKD4	22 (0.1%)	7 (0.1%)	0 (0.0%)	5 (0.1%)	10 (0.1%)	
CKD5	9 (0.0%)	2 (0.0%)	3 (0.1%)	0 (0.0%)	4 (0.0%)	
Laboratory biomarker						
Serum calcium, mmol/L	2.3 ± 0.1	2.3 ± 0.1	2.3 ± 0.1	2.3 ± 0.1	2.4 ± 0.1	<0.001
ALT, U/L	24.4 ± 17.8	26.3 ± 22.1	25.8 ± 19.8	23.7 ± 14.9	23.1 ± 15.8	<0.001
AST, U/L	24.5 ± 13.7	25.4 ± 16.7	24.6 ± 18.2	23.8 ± 10.3	24.4 ± 10.4	<0.001
Scr, umol/L	75.0 ± 19.9	72.0 ± 20.0	73.8 ± 24.4	75.5 ± 16.8	76.9 ± 18.6	<0.001
Albumin, g/L	42.9 ± 3.4	42.2 ± 3.5	42.9 ± 3.3	43.2 ± 3.3	43.0 ± 3.5	<0.001
Triglyceride, mmol/L	1.6 ± 1.4	1.6 ± 1.7	1.7 ± 1.6	1.6 ± 1.2	1.5 ± 1.3	<0.001
Cholesterol, mmol/L	5.0 ± 1.1	4.9 ± 1.1	5.0 ± 1.0	5.0 ± 1.0	5.1 ± 1.1	<0.001
Total bilirubin, umol/L	11.3 ± 5.5	10.7 ± 5.6	11.2 ± 5.4	11.4 ± 5.3	11.5 ± 5.6	<0.001
BUN, mg/dL	12.9 ± 4.4	11.3 ± 4.0	12.5 ± 4.1	13.1 ± 4.1	13.8 ± 4.6	<0.001
sUA, umol/L	310.7 ± 79.0	313.8 ± 83.2	318.0 ± 80.9	313.6 ± 76.6	301.9 ± 76.7	<0.001
HbA1c, %	5.5 ± 0.8	5.6 ± 1.0	5.5 ± 0.9	5.5 ± 0.7	5.4 ± 0.6	<0.001
GFR, mL/min/1.73 m^2^	99.5 ± 20.0	108.5 ± 20.1	104.1 ± 18.7	99.1 ± 18.5	92.1 ± 19.1	<0.001
Higher systolic blood pressure, (%)	3,382 (16.6%)	857 (18.5%)	769 (14.9%)	850 (16.0%)	906 (17.1%)	0.006
Higher diastolic blood pressure, (%)	2,739 (15.6%)	716 (16.5%)	722 (17.2%)	672 (15.0%)	629 (14.7%)	0.033
Higher blood pressure, (%)	4,769 (25.0%)	1,204 (26.7%)	1,153 (24.7%)	1,182 (24.1%)	1,230 (25.1%)	0.177

BMI, body mass index; CAD, coronary artery disease; CKD, chronic kidney disease; ALT, alanine aminotransferase; AST, aspartate aminotransferase; Scr, serum creatinine; BUN, blood urea nitrogen; sUA, serum uric acid; GFR, glomerular filtration rate.

### 3.2. Correlations between serum 25(OH)D concentrations and BP

As previously stated, we built three logistic models to explore the connection between serum 25(OH)D concentrations and BP (Table 2).

**Table 2:** The association between serum 25(OH)D and blood pressure.

**Exposure**	**Model 1**			**Model 2**			**Model 3**		
	**OR (95% CI)**	***p***	***p* trend**	**OR (95% CI)**	***p***	***p* trend**	**OR (95% CI)**	***p***	***p* trend**
Higher blood pressure									
Serum 25(OH)D, nmol/L									
<44.3	Ref		0.280	Ref		<0.001	Ref		0.096
44.3– <60.5	0.90 (0.80, 1.02)	0.086		0.86 (0.76, 0.99)	0.029		0.90 (0.78, 1.05)	0.185	
60.5– <77.7	0.87 (0.77, 0.98)	0.023		0.75 (0.65, 0.85)	<0.001		0.85 (0.72, 0.99)	0.041	
≥77.7	0.92 (0.81, 1.04)	0.184		0.69 (0.59, 0.79)	<0.001		0.86 (0.72, 1.02)	0.085	
Higher systolic blood pressure									
Serum 25(OH)D, nmol/L									
<44.3	Ref		0.699	Ref		<0.001	Ref		0.055
44.3– <60.5	0.77 (0.66, 0.90)	0.001		0.75 (0.63, 0.89)	0.002		0.79 (0.65, 0.96)	0.016	
60.5– <77.7	0.84 (0.73, 0.97)	0.017		0.71 (0.60, 0.84)	<0.001		0.82 (0.67, 1.00)	0.046	
≥77.7	0.91 (0.79, 1.04)	0.160		0.62 (0.52, 0.73)	<0.001		0.78 (0.63, 0.96)	0.018	
Higher diastolic blood pressure									
Serum 25 (OH)D, nmol/L									
<44.3	Ref		0.022	Ref		0.001	Ref		0.275
44.3– <60.5	1.05 (0.92, 1.21)	0.463		1.01 (0.88, 1.17)	0.840		1.05 (0.89, 1.25)	0.543	
60.5– <77.7	0.89 (0.78, 1.02)	0.103		0.82 (0.71, 0.95)	0.007		0.90 (0.76, 1.08)	0.261	
≥77.7	0.87 (0.74, 1.03)	0.099		0.78 (0.66, 0.94)	0.008		0.93 (0.75, 1.16)	0.502	

Model 1 was unadjusted. Model 2 was adjusted for age, gender, and ethnicity. Model 3 was adjusted for age, gender, ethnicity, education level, smoking status, heavy alcohol drinking, vitamin D supplementation, BMI, waist circumference, diabetes, coronary heart disease, stroke, cancer, sleeplessness, CKD, serum calcium, ALT, AST, Scr, albumin, triglyceride, cholesterol, total bilirubin, BUN, sUA, and HbA1c.

As for higher BP, in Model 1, which was unadjusted for variables, the HRs (95% CI) for the Q2 (44.3–60.5), Q3 (60.5–77.7), and Q4 (≥77.7) were 0.90 (0.80, 1.02), 0.87 (0.77, 0.98), and 0.92 (0.81, 1.04), respectively, compared with the Q1 (<44.3). After adjusting for age, gender, and ethnicity in Model 2, the HRs (95% CI) of higher BP for Q2, Q3, and Q4 were 0.86 (0.76, 0.99), 0.75 (0.65, 0.85), and 0.69 (0.59, 0.79), respectively, compared with the reference (Q1). Meanwhile, in Model 3, compared with Q1, the HRs (95% CI) for Q2, Q3, and Q4 were 0.90 (0.78, 1.05), 0.85 (0.72, 0.99), and 0.86 (0.72, 1.02) after adjusted for multiple confounders.

We further evaluated the effect of serum 25(OH)D concentrations on higher systolic and diastolic blood pressure alone. In Model 3, Q2-4 had a significantly lower HR (95% CI) of higher systolic BP than the reference (Q1). However, opposite of Q3 (60.5–77.7) and Q4 (≥77.7), Q2 (44.3–60.5) tended positive correlation with higher diastolic BP in Model 3, but it did not reach statistical significance (*p* > 0.05).

### 3.3. Restricted cubic spline analyses

**Figure 2:**
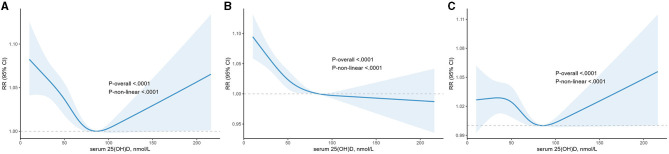
The dose-response analysis between serum 25(OH)D and higher blood pressure, higher systolic blood pressure and higher diastolic blood pressure with restricted cubic splines. The solid blue line and light blue area represent the estimated odds ratios and its 95% confidence interval, respectively. **(A)** Higher blood pressure; **(B)** higher systolic blood pressure; **(C)** higher diastolic blood pressure.

To address non-linearity, restricted cubic spline analyses were used to visualize the association of serum 25(OH)D concentrations and BP, indicating a U-shaped correlation (*p* for non-linear <0.0001, Figure 2A). Meanwhile, an approximate L-shaped relationship was found between serum 25(OH)D and elevated SBP alone (p for non-linear =0.0009, Figure 2B). However, a complex relationship, roughly Z-shaped, was found between serum 25(OH)D and DBP (p for non-linear =0.0064, Figure 2C).

### 3.4. Subgroup analysis

**Figure 3:**
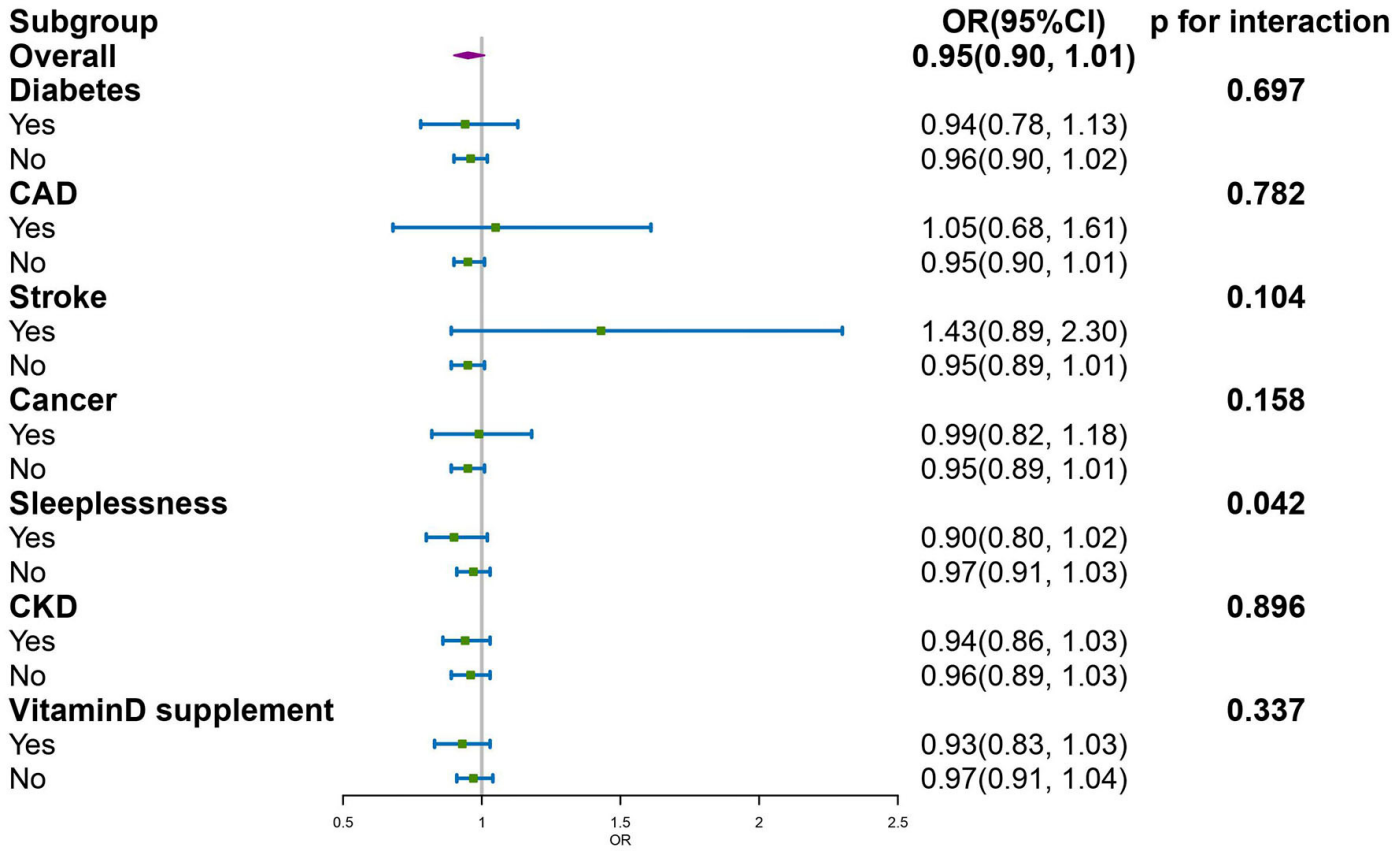
Subgroup analyses of the serum 25(OH)D and higher blood pressure.

We adjusted the confounding factors in Model 3 in these subgroups. CKD1 was considered to have no chronic kidney disease (CKD), and CKD2 to CKD5 were regarded as having chronic kidney diseases. As shown in Figure 3, the effect of serum 25(OH)D on blood pressure is consistent across the subgroups except for sleeplessness (*p* for interaction < 0.05).

## 4. Discussion

Our analyses, based on a nationally representative population from seven consecutive NHANES cycles (2005–2018), were performed to explore the relationship between serum 25(OH)D concentrations and BP in adults with no previous diagnosis of hypertension in the US. The key findings could be summarized in three points. First, after adjusting multiple variables, serum 25(OH)D was negatively correlated with BP, primarily SBP. Second, the relationships between serum 25(OH)D concentration and BP, including SBP and DBP, were non-linear. Third, the relationship was consistent across the subgroups except for sleeplessness.

Early in the 1980s, Sowers et al. (8) discovered a significant negative relationship between ingestion of vitamin D and BP. In the following decades, cross-sectional studies based on the Third National Health and Nutrition Examination Survey (NHANES III) have obtained the same conclusions (9, 10). However, prospective cohort studies elicited inconsistent results. After 4 years of follow-up, Forman et al. (11) found that compared with women without vitamin D deficiency [25(OH)D <15 ng/ml], women with vitamin D deficiency had a multivariable relative risk (RR) of 2.98 (95% CI: 1.24 to 7.20). Moreover, the risk was even more markedly elevated among men with vitamin D deficiency. Nonetheless, after an 8-year follow-up period, the RRs for hypertension incidents had reduced and were no longer statistically significant. It is noteworthy that there are only 33 participants with 25(OH)D deficiency in this study, so the representativeness of its outcomes needs to be queried. In the Tromsø study conducted by Jorde et al. (12), they proceeded with a 14-year follow-up from 1994 to 2008. They observed that, after controlling variables, serum 25(OH)D in 1994 failed to predict future hypertension or increased BP, and that there was no significant relationship between changes in serum 25(OH)D and changes in BP from 1994 to 2008. Moreover, various researchers had further investigated the relationship between vitamin D supplementation and lowering BP, obtaining negative (13–17) and positive (18–21), seemingly contradictory results, which brought into doubt the prospects of vitamin D for lowering BP. But previous studies had some limitations: small sample size (11), not taking into account some important covariates (such as chronic disease (9, 10, 12), smoking/drinking (10, 11), dietary supplement use (9, 11, 12), etc., which could significantly influence serum 25(OH)D status and prevalence of hypertension), specific participants population. However, our research was a significant cross-sectional study and employed a nationally representative sample of US adults, excluding those taking hypotensive drugs or history of physician-diagnosed hypertension participants to minimize the likelihood that they improve their BP through lifestyle changes and agents. At the same time, we adjusted for lifestyle habits, comorbidities, and serological indicators and further analyzed the effect of serum 25(OH)D concentration on systolic and diastolic blood pressure, respectively. Consistent with the previous cross-sectional studies, we found a negative relationship between serum 25(OH)D levels and higher BP. At the same time, our study further revealed that higher serum 25(OH)D levels were associated with lower SBP. As for the contribution between serum 25(OH)D levels and DBP, there was even a positive effect, but it was not statistically significant.

So far, the exact mechanism of hypotension by serum 25(OH)D is still unknown, and there are several possible core factors. An animal experiment conducted by Li et al. (22) found a several-fold increase in renin expression and plasma angiotensin II production in mice with vitamin D receptor-null (VDR-null). Thus, it gave rise to the earliest and currently widely accepted mechanism that vitamin D can lower BP by suppressing renin synthesis to downregulate the activity of the renin-angiotensin-aldosterone system (RASS) (23). Moreover, vitamin D inhibits the production of parathyroid hormone (PTH), which can increase BP by stimulating PTH2 receptors expressed on vascular smooth muscle cells, upregulating both receptors of advanced glycation end products (RAGE) expression and monocyte-macrophages cytokines and IL-6 production, and promoting the deposition of calcium in the arterial wall leading to increasing collagen deposition and vessel stiffness (24). These imply that lack of vitamin D may be a risk factor for hypertension. In our study, when serum 25(OH)D <85 nmol/L, it was negatively correlated with SBP but not with DBP. A cross-sectional study of the biethnic population discovered that SBP was inversely associated with 25(OH)D levels in whites but not in blacks (25). Later on, Jafari et al. (26) conducted a meta-analysis of 26 randomized controlled trials for patients with diabetes and demonstrated that vitamin D improved SBP in type 2 diabetic patients. Subsequent studies have revealed similar findings (27–29), but few studies have been conducted in patients without hypertension, and our experiment fills this gap. It is well known that BP is defined as the lateral pressure exerted on the walls of blood vessels per unit area during the flow of blood (3). Part of the stroke volume is sent directly to peripheral tissues during ventricular contraction, resulting in systolic pressure, while part of it is temporarily stored in the aorta and central artery, stretching the arterial wall and rising local BP, resulting in diastolic pressure (30). BP is directly influenced by two primary factors: the volume of intravascular fluid and the capacity for vasodilation. As previously mentioned, vitamin D can downregulate the activity of the RASS, which exerts various physiological and pathophysiological effects including vasoconstriction and sodium/water retention to elevate BP *via* activation of angiotensin II type 1 receptor (AT1R). In addition, when vitamin D deficiency induces vascular sclerosis and stretching disturbances, the entire stroke volume flows through the arterial system and peripheral tissues, which results in increased SBP and decreased DBP (30). Further investigations of the antihypertensive mechanism of vitamin D are now warranted.

Increasing evidence supports the existence of a threshold effect for vitamin D levels (31). In our study, as mentioned previously, the relationship of vitamin D with higher blood pressure and higher SBP was U- and L-shaped, respectively. Furthermore, both of them had a cut-off value of approximately 84 nmol/L. For higher blood pressure, the risk was increased for both vitamin D levels below or above 84 nmol/L. The risk of higher SBP rose significantly below 84 nmol/L but remained marginally significant above 84 nmol/L. These outcomes indicated that subjects with vitamin D insufficiency or deficiency were more sensitive to vitamin D supplementation, and there was a risk of higher blood pressure, primarily DBP, when vitamin D is above 84 nmol/L. In support, previous studies also showed that the antihypertensive effect of vitamin D supplementation was observed only in patients with vitamin D insufficiency or deficiency (21, 32–34). However, to the best of our knowledge, a particular Z-shaped relationship between serum 25(OH)D and DBP was detected for the first time. It revealed a negative relationship between serum 25(OH)D and DBP within the range of 36 to 84 nmol/L but a positive relationship below or above the range.

Upon further subgroup analysis, we found that the inverse association between serum 25(OH)D and BP tends to be more pronounced and sustained among participants with sleeplessness. As well, recently, a cross-sectional study showed a more pronounced and stable association between vitamin D status and coronary heart disease in participants with poor sleep patterns (35). As far as we know, sleeplessness is recognized as short sleep duration and insomnia, and daytime sleepiness may be linked to reduced outdoor exercise and sunlight exposure, which may diminish serum vitamin D *via* endogenous synthesis (36, 37) and boost melatonin expression (38). As a result, subjects with sleeplessness had lower levels of serum vitamin D compared with those without sleeplessness, so the decline in blood pressure resulting from serum vitamin D levels was more evident according to our findings. However, further studies are needed to validate these findings.

It is important to note that there are some limitations in our study. First, there is a dearth of data on participants' salt intake and sun exposure, which are important factors in endogenous vitamin D production and hypertension, respectively. Second, we performed a single measurement of plasma 25(OH)D, which had a marked circadian variation. Third, because BP was measured merely in a single appointment, it is possible that some participants were wrongly labeled as having higher blood pressure while they were actually normotensive, and vice versa. Fourth, biases resulting from excluded and unmeasured confounders may still exist, despite our efforts to account for as many confounders as possible. Fifth, relationships between 25(OH)D and BP stratified by sleeplessness deserve to be further verified. Finally, the cross-sectional design also makes it impossible to distinguish between causes and effects.

## 5. Conclusion

We discovered a non-linear association between serum 25(OH)D concentration and BP in a nationally representative sample of US participants without a previous diagnosis of hypertension. Concentrations of 25(OH)D were inversely associated with blood pressure when it was <84 nmol/L. More specifically, serum 25(OH)D is adversely correlated with systolic blood pressure, and as for diastolic blood pressure, higher serum 25(OH)D concentrations were associated with higher diastolic blood pressure when serum 25(OH)D levels >84 nmol/L.

## Data availability statement

Publicly available datasets were analyzed in this study. This data can be found here: https://www.cdc.gov/nchs/nhanes/index.htm.

## Ethics statement

The studies involving human participants were reviewed and approved by the National Center for Health Statistics Research Ethics Review Board. The patients/participants provided their written informed consent to participate in this study.

## Author contributions

JC: Writing—original draft, Investigation, Validation. JT: Validation, Writing—original draft. XK: Data curation, Formal analysis, Writing—review and editing. CZ: Data curation, Formal analysis, Writing—review and editing. RZ: Visualization, Writing—review and editing. JS: Visualization, Writing—review and editing. XZ: Visualization, Writing—review and editing. ZL: Conceptualization, Writing—review and editing.
